# Supplementary material for: Mussel periostracum protects against shell dissolution
Source: PLoS One. 2025 Jul 16;20(7):e0327170. doi: 10.1371/journal.pone.0327170 (PMC12266434; doi:10.1371/journal.pone.0327170)
Supplement: S1 Table — We measured total alkalinity and pH in order to estimate the remaining seawater parameters, along with salinity and temperature. We report calcium carbonate saturation state values for the both aragonite and calcite mineral forms but note the higher presence of aragonite mineral in Mytilus shells relative to the calcite mineral form. (DOCX) [file pone.0327170.s001.docx]

**SUPPLEMENTARY MATERIAL—**

Table S1. Description of the full set of carbonate system conditions for each of the laboratory experiments. We measured total alkalinity and pH in order to estimate the remaining seawater parameters, along with salinity and temperature. We report omega values for the aragonite calcium carbonate mineral form because of its higher presence in *Mytilus* shells relative to the calcite mineral form. Column labels are as follows: Experiment ID, temperature (°C), salinity, total alkalinity (µmol kg^-1^), pH (total scale), partial pressure of carbon dioxide (pCO_2_, µatm), dissolved inorganic carbon (DIC, µmol kg^-1^), saturation state of the aragonite (Ω_aragonite_) and calcite (Ω_calcite_) mineral forms of calcium carbonate. Values are listed as the mean ± standard deviation for each experiment. Sample size is listed for each treatment condition (n = sample size).

| **Experiment** | **Temp. (°C)** | **Salinity** | **TA**  **(µmol kg^-1^)** | **pH**  **(total scale)** | **pCO_2_**  **(µatm)** | **DIC**  **(µmol kg^-1^ )** | **Ω_aragonite_** | **Ω_calcite_** |
| --- | --- | --- | --- | --- | --- | --- | --- | --- |
| Periostracum cover  (n = 49) | 10.8 ± < 0.01 | 34.13 ± < 0.01 | 2306 ± 9 | 7.51 ± 0.01 | 1539 ± 50 | 2307 ± 10 | 0.7 ± 0.02 | 1.0 ± 0.02 |
| pH  (n_7.7_ = 9)  (n_7.4_ = 16) | 9.9 ± < 0.01 | 33.89 ± < 0.01 | 2270 ± 8 | 7.74 ± 0.01 | 868 ± 13 | 2207 ± 8 | 1.0 ± 0.01 | 1.6 ± 0.01 |
|  | 10.2 ± < 0.01 | 33.92 ± < 0.01 | 2277 ± 1 | 7.38 ± 0.01 | 2060 ± 64 | 2319 ± 1 | 0.5 ± 0.02 | 0.8 ± 0.02 |
| Sanding  (n = 23) | 10.2 ± < 0.01 | 33.94 ± < 0.01 | 2283 ± 15 | 7.41 ± 0.01 | 1954 ± 29 | 2318 ± 15 | 0.5 ± 0.01 | 0.8 ± 0.01 |
