# Supplementary material for: Mussel periostracum protects against shell dissolution
Source: PLoS One. 2025 Jul 16;20(7):e0327170. doi: 10.1371/journal.pone.0327170 (PMC12266434; doi:10.1371/journal.pone.0327170)
Supplement: Attachment — Submitted filename: Response to Reviewers_Pt2.docx.pdf [file pone.0327170.s003.pdf]

Response to Reviewers

We thank the two reviewers for their careful evaluation of our manuscript. We have clarified the chemical protective role of the periostracum, better defined the physical and biological agents involved in periostracum removal, and expanded explanations on the importance of temperature and salinity measurements for accurate seawater carbonate analysis. These revisions have enhanced the clarity of our study, and we appreciate the reviewer's insights.

Below, we respond (in italicized text) to each comment in sequence, referencing the relevant line number of the revised manuscript.

Sincerely,

Dr. Alisha Saley

<u>Reviewer #2:</u>

The corrections and explanations provided were well-written and cohesive. The additional information added enhanced the context and improved the readability of the paper, leading to a better understanding. However, it would be beneficial to include some further details, as suggested below, so minor revision are needed. Overall, excellent work and writing, the revisions made were well-done.

Really good explanations were added, adding more context made the abstract clearer.

We are pleased that Reviewer #2 supports the corrections made to improve the explanations, context, and readability of our paper. We appreciate the kind remarks regarding our efforts.

Is it possible to add whether the periostracum has any chemical capacity against the acidic conditions?

We thank the reviewer for encouraging us to clarify our wording regarding the potential for the periostracum to chemically shield the underlying shell from corrosive seawater conditions. We now more clearly explain that the 'chemical capacity against acidic conditions' refers to the hydrophobic nature and low porosity of the periostracum's layered structure—i.e., the chemical properties of the periostracum material itself, rather than chemical reactions between the periostracum and seawater—as a means of protecting the underlying shell (lines 67–68, 74). While we do not speculate on possible chemical interactions between the periostracum and seawater, as this lies beyond the scope of our study, we emphasize the value of future research exploring this line of inquiry.

The explanation added is relevant and good, but the phrasing could be slightly better to add clarity (Lines 73-76). Even though the information added is helpful, you could clarify the composition of the periostracum varies among different mussels species in this part (Lines 78-79).

We appreciate the reviewer's request to clarify that periostracum thickness, porosity, and organic composition are characteristics that may influence the extent of shell corrosion. In our revised explanation, we also note that these characteristics can vary among mussel species (lines 75–77).

Adding the specific physical agents found in intertidal habitats that you explored would be good in the first part. For the discussion part, maybe adding further examples of how predation and microbial activity interact with physical agents in the removal of periostracum would be good too (Lines 107-109; 568-569).

We thank the reviewer for highlighting the need to better define the physical agents examined in our study. We have added further clarification for the reader (lines 109–110, 114–115). Additionally, we included text clarifying that predation and parasitic activity (i.e., biological agents of removal) may interact with, and be influenced by, physical agents in the removal of the periostracum layer (lines 495, 505–510).

The link between temperature, salinity and alkalinity measurement could be made clearer (Line 190-195).

We thank the reviewer for encouraging us to clarify the importance of measuring temperature and salinity to accurately analyze total alkalinity and subsequently calculate other parameters in the seawater carbonate system (lines 181–182). We have explained how temperature and salinity influence seawater pH and emphasized their critical role in the accurate measurement and calibration of spectrophotometric methods (lines 189–191). Additionally, we included a brief description of how alkalinity is analyzed via acid titrations (lines 202–205). Finally, we provide a more detailed explanation of the relationship between salinity and alkalinity and why precise salinity measurements are essential for calculating alkalinity from a titration curve (lines 205–211).

Good adding (Lines 498-500).

We are grateful to the reviewer for their supportive remarks.

Reviewer #3: (no response)
