# Supplementary material for: Mussel periostracum protects against shell dissolution
Source: PLoS One. 2025 Jul 16;20(7):e0327170. doi: 10.1371/journal.pone.0327170 (PMC12266434; doi:10.1371/journal.pone.0327170)
Supplement: Attachment — Submitted filename: Response to Reviewers.docx [file pone.0327170.s002.docx]

**Response to Reviewers**

We thank the two reviewers for their careful evaluation of our manuscript. The suggestions provided have significantly improved the clarity of presentation of our research findings, and we are pleased to have been able to follow nearly every recommendation.

Below, we respond (in italicized text) to each comment in sequence, referencing the relevant line number of the revised manuscript.

Sincerely,

Dr. Alisha Saley

**Reviewer #1:**

In this study, the authors provided evidence that the periostracum helps mussels protect against shell dissolution. I find this paper to be an interesting addition to the understanding of the protective abilities of bivalves against ocean acidification and believe it is publishable in PLOS ONE. However, the paper requires revision due to several uncertainties that need clarification before publication. Specifically, it was difficult to determine how the periostracum cover influenced the dissolution rate. I would recommend including sample images of the periostracum and the dissolved surfaces of mussels.

*We are grateful to Reviewer #1 for their positive remarks concerning the value of our study. We concur that more detail regarding imaging the periostracum would be helpful, and now include an additional figure that more clearly demonstrates the contrast between the periostracum and the underlying shell in this species (new Fig. 1, lines 168-172). We also include more language describing ways in which the periostracum has been believed to influence shell dissolution. Although these concepts were largely untested prior to our study, they outline general thinking on the topic (Lines 472-474, 486-489)*

Which of the two valves was used? Depending on the species, the two valves may not be symmetrical.

*Mytilus mussels are bilaterally symmetrical, such that the left and right valves have near-equivalent size and shape. We now state this information for the reader (Lines 158-160).*

Is it possible to see the periostracum with the naked eye? What criteria were used to identify the periostracum? Please provide additional details in the supplementary material. It was difficult to understand how varying percentages of periostracum cover influenced the dissolution rate. Could you provide sample images for each end of the spectrum? This would make it much clearer.

*We thank the reviewer for posing these three related questions. In* Mytilus californianus*, the periostracum is visible to the naked eye, appearing as a dark brown to black covering over a lighter grey to light bluish purple that characterizes the underlying shell. These color differences are reinforced by discrete edges to the periostracum in locations where it is discontinuous. We now clarify these visible criteria, and add a new figure that shows images before and after delineating periostracum from the underlying shell, including a range of coverage percentages (new Fig. 1, lines 168-172).*

Why do mussels abraded with fine sand show a negative dissolution rate (lower than controls)?

*In Fig 5 (originally Fig 4), dissolution rates are plotted relative to a periostracum-free control shell, where y = 0 indicates an equivalent dissolution rate to controls. Thus, negative dissolution rates indicate that when shells were abraded with fine grit, the measured shell loss was less than that of unabraded control shells. We now streamline the pertinent phrasing in the text and highlight this point further in the figure caption (lines 424-426). In terms of the mechanistic basis for this pattern, we suspect it may be due to reduced surface roughness (and thus less total surface area at the microscopic level that could contribute to dissolution) in shells abraded with the fine sand.*

Is there a specific reason for not using SEM? SEM offers much higher resolution and could provide more precise results. Why wasn’t the periostracum thickness measured, even though it is important?

*We appreciate the reviewer inquiring about using scanning electron microscopy or other high-resolution techniques to measure the periostracum and its thickness. Unfortunately, although we agree with the reviewer that such methods would have been appropriate and potentially informative, SEM was not readily available to us. We emphasize that we are confident that our digital imaging technique is robust and adequate to characterize the broad scope of variation in periostracum cover observed in our mussels*. *We do now note in the text that future research could use higher resolution instrumentation to follow up on our work (lines 533-536).*

Reviewer #2:

This study has the objective to investigate the impact of periostracum coverage on the ability of California mussels to resist shell dissolution under a diverse environment stressors, especially ocean acidification, where the authors explored how shell size, age and environmental conditions (such as food availability and wave exposure) can interact with the periostracum to influence dissolution rates. The paper is very well written, well-organized and insightful, providing clear explanations of the experimental design and results.

*We are grateful to the reviewer for their supportive remarks.*

You can change “possibility” to “hypothesis”. You can change “Because” to “Since”. If you have enough space, you should specify the meaning of “rougher surface texture” and its effects on dissolution.

*We agree with the word change suggestions and have amended accordingly (lines 31, 34). We also appreciate the request to clarify surface texture and have added text to explain how rougher textures may link to higher dissolution rates (lines 39-43).*

How the desiccation at low tide can contribute to damage?

*We thank the reviewer for encouraging us to explain the connection between low tide and periostracum damage. We now clarify better that desiccation and temperature cycling at low tide could weaken the periostracum’s attachment to the underlying shell, which may result in higher amounts of detachment (lines 43-46).*

Add how mussels modify local physical factors and its impacts (water flow, sediment stability, etc).

*We are grateful for the request to add more details as to how mussels modify local factors and have amended the text to include the specific factors explored in the referenced studies (lines 58-60).*

On mechanical and chemical armour, does it mean periostracum will prevent shell thinning? Or does it neutralize acidic conditions?

*We thank the reviewer for highlighting the need to better define ‘armour’ from the referenced study. The periostracum acts as both a mechanical and chemical barrier, protecting the shell from seawater conditions that may encourage dissolution. We now clarify this meaning for the reader (lines 68-70).*

Why do particles attached reduce OA-associated dissolution?

*We appreciate the question for highlighting the need to elaborate on how clay particles around the periostracum and shell may reduce dissolution in corrosive seawater. The referenced study discusses how clay shields protect gastropod shells in low-pH estuaries by creating a low-permeability barrier. We’ve added further clarification for the reader (lines 73-76)*.

What are the implications [*of periostracum thickness and composition*]? Are there variations in the chemical compositions among different mussels species that can affect its ability to withstand OA?

*We agree with the request to expand on the implications of periostracum thickness and composition in dictating the degree of shell corrosion in low pH seawater. We now clarify more explicitly how these traits influence the severity of corrosion by highlighting their potential to mediate contact between seawater and the underlying shell (lines 78-79)*.

You can change “may propagate” to “may disrupt”.

*We appreciate the suggestion; however, we believe the change would alter the intended meaning. Instead, we have revised the text to emphasize the significance of understanding how individual responses cascade to influence relationships among species (lines 87).*

Change “not living tissue” to “non-living tissue”.

*We appreciate this suggestion and have amended accordingly (line 88).*

What are the molecular mechanisms in response to acidification? Microbial activity impacts or influences the degradation of periostracum?

*We agree that additional molecular studies and explorations of microbial effects would be valuable, but such efforts are outside the realm of our study.*

How periostracum degradation varies across different intertidal zones/ecosystems? Does UV radiation have any impact/role on structural integrity? What are the long-term effects of tidal exposure?

*We appreciate the note on variability in mechanisms driving degradation in intertidal habitats and the suggestion to include UV radiation as a factor influencing periostracum integrity. Additionally, we appreciate the request for clarification on effects of tidal exposure. We now expand on these connections in line 99.*

Change “undertook” to “conduct”.

*We have amended the phrasing as suggested (line 105).*

Is it possible to identify the specific conditions where the periostracum loss accelerates?

*We appreciate the question; however, due to our experimental design testing only three discrete pH conditions, we are unable to discern whether dissolution accelerates in a non-linear fashion outside our tested range. We now highlight this point more clearly in the discussion section to improve transparency of experiment limitations (lines 494, 498).*

Predation or microbial actions are most effective in removing periostracum?

*We agree with the reviewer that predation and microbial activity are likely effective agents in the removal of the periostracum. Given other research on these pathways, we sought to explore additional, untested mechanisms, including physical agents found in intertidal habitats, but we do emphasize the importance of both physical and biological factors later in the discussion (lines 107-109, 568-569).*

Capitalize the word “species” -> Species.

*We appreciate the suggested change, however we must leave headers in sentence case to comply with PLOS One guidelines for header naming.*

Do the environmental factors in these regions have any influence on their reproductive patterns? What are the key factors that can influence the diversity and survival of the species?

*We thank the reviewer for drawing attention to variability in reproduction, diversity and survival being driven by regional environmental heterogeneity. These topics are broad and beyond what we could hope to examine in our study, but we do now highlight more overtly that reproductive state is a potential influence on energetic investment to morphology (lines 546-548).*

Add a coma after 2022.

*We have made the change as requested (Line 271).*

Capitalize the word “experiments” -> Experiments.

*We appreciate the suggested change, however we must leave headers in sentence case to comply with PLOS One guidelines for header naming*

Is there any notable variation based on the region of collection?

*We thank the reviewer for highlighting that mussel traits may vary based on collection location. We agree and clarify that collections were performed at a single location to control for the influence of site (lines 129-130).*

Age, shell integrity or any other physiological factors can have any influence on the dissolution rates in the shells used in these trials?

*We are grateful to the reviewer for highlighting the potential influence that age and shell integrity may have on dissolution rates of mussels used in this trial. We agree, and have attempted to highlight these factors more clearly in the discussion (lines 513-515).*

What interactions between the factors were noticed?

*We thank the reviewer for this question as it highlights a need to better clarify our experimental design and lack of capacity to test the interactions between some factors. We have included additional text to better clarify the independence of the tests listed (lines 148-149). In particular, we tested the influence of periostracum and pH as an interaction in the second lab experiment and omitted the term in the final model as it was not significant (lines 305-306). We did not test the interaction between periostracum cover and shell roughness, as all periostracum was removed in roughness examinations, however, follow up research could include iterative removal of periostracum using varying grit sizes to determine the extent of an interaction.*

Capitalize “Effects of periostracum cover” -> Effects of Periostracum Cover.

*We appreciate the suggested change, however we must leave headers in sentence case to comply with PLOS One guidelines for header naming.*

How can seasonal variations influence the durability of periostracum across different habitats?

*We are unaware of prior research investigating the influence of seasonal conditions on periostracum durability but agree that this could be an interesting next step in investigations. As a non-living tissue, we highlight how environmental conditions may have significant influence on periostracum durability during initial deposition (Lines 532-541).*

How do the factors (temperature, salinity) interfere with accurate alkalinity measurements?

*We thank the reviewer for highlighting the influence of temperature and salinity on alkalinity measurements. We agree that controlling these properties is crucial for accurate reporting and have followed standard practices in measuring and estimating carbonate system parameters, as noted on lines 190-195).*

Change “rate” to “rates”. Can change “establishment of any chemical” to “formation of chemical gradients”.

*We agree with the word change suggestions and have amended accordingly (lines 155, 193).*

What effects can appear from any slight chemical gradient formation?

*We thank the reviewer for the question on chemical gradient influence in seawater treatment. We’ve added a clearer explanation of what would occur without bottle agitation during the experiment. (lines 193-194).*

Why were these pH levels chosen? Do they correlate to natural conditions?

*We originally listed the reason for the additional two pH levels chosen, but have moved the explanation earlier in the text to highlight it more lines 208-212).*

How shifts can impact different species across global environments?

*We appreciate the reviewer drawing attention to how our work may inform research of other mollusc species. We have noted this more explicitly in our discussion and highlight the importance of additional work in more taxa (lines 484-485).*

Capitalize “seawater” -> Seawater.

*We appreciate the suggested change, however we must leave headers in sentence case to comply with PLOS One guidelines for header naming*

Why were these two techniques chosen?

*We clarified that these standard techniques for creating ocean acidification conditions increase dissolved inorganic carbon (DIC) concentrations—either by adding CO2 gas, that reacts with seawater and enters the carbonate system as DIC, or chemically—without altering alkalinity, leading to a pH decline. We previously mentioned equipment unavailability as the reason for using two separate but chemically equivalent methods and have now moved this explanation earlier in the methods for clarity (lines 245-247).*

How the variation of CO2 concentration in natural environments can affect the carbonic acid equilibrium?

*We appreciate the request to specify the result of variation in carbonate system conditions in the coastal environment. We have chosen to reference the resultant variability as pH because it is most relevant to abiotic shell dissolution, though we acknowledge that the system can also be described as DIC, pCO2 or TA (lines 496-497, 142-147).*

The type of habitat (sheltered, exposed microhabitats) will affect the periostracum coverage in natural mussel populations? How?

*We agree with the reviewer that the level of exposure for the microhabitat overall will influence the degree of periostracum cover in natural mussel populations. We have attempted to clarify and highlight this point in the discussion (lines 502-504).*

How the methods used to normalize dissolution rates will influence the outcomes?

*We appreciate the question on our statistical model choice and have clarified how normalizing dissolution rates by shell size controls for its potential influence on the response measurement. We also added more detail on the square root transformation of the response variable to meet linearity assumptions for the model (lines 286-290).*

Add comma after effect. Change “residual” to “residuals”. Remove “a” before “Welch’s”.

*We agree with the word change suggestions and comma additions, and have amended accordingly (lines 292-296, 313)*

Capitalize “cover and shell loss” -> Periostracum Cover and Shell Loss.

*We appreciate the suggested change, however we must leave headers in sentence case to comply with PLOS One guidelines for header naming*

How does the loss of periostracum in areas with more sun activity interact with their ability to survive? Since the ocean temperature is increasing.

*We are grateful for the recommendation to highlight the influence of increasing temperature of air at low tide, and in seawater, on mussel survival. Though we recognize that extreme heat events occur and cause large die-off events, we have included this point in terms of temperature stress and not explicitly survival (lines 562-564).*

Add comma after degradation.

*We appreciate the suggestion to improve clarity. Instead of adding a comma, we’ve revised the text to better explain that spots of degradation facilitate further detachment (lines 498-500).*

How will the increased exposure impact the ability to adapt?

*We appreciate the focus on the adaptive capacity of mussels experiencing shell damage under corrosive seawater conditions. We have highlighted this issue as an important area for future research (lines 525-528).*
